# Reference genes for accurate gene expression analyses across different tissues, developmental stages and genotypes in rice for drought tolerance

**DOI:** 10.1186/s12284-016-0104-7

**Published:** 2016-07-18

**Authors:** Isaiah M. Pabuayon, Naoki Yamamoto, Jennylyn L. Trinidad, Toshisangba Longkumer, Manish L. Raorane, Ajay Kohli

**Affiliations:** Genetics & Biotechnology Division, International Rice Research Institute, DAPO 7777, Metro Manila, 1301 Philippines

**Keywords:** Drought, Gene expression, qRT-PCR, Reference gene, Rice, Transcriptome

## Abstract

**Background:**

Quantitative reverse transcription PCR (qRT-PCR) has been routinely used to quantify gene expression level. This technique determines the expression of a target gene by comparison to an internal control gene uniformly expressed among the samples analyzed. The reproducibility and reliability of the results depend heavily on the reference genes used. To achieve successful gene expression analyses for drought tolerance studies in rice, reference gene selection should be based on consistency in expression across variables. We aimed to provide reference genes that would be consistent across different tissues, developmental stages and genotypes of rice and hence improve the quality of data in qRT-PCR analysis.

**Findings:**

Ten candidate reference genes were screened from four ubiquitously expressed gene families by analyzing public microarray data sets that included profiles of multiple organs, developmental stages, and water availability status in rice. These genes were evaluated through qRT-PCR experiments with a rigorous statistical analysis to determine the best reference genes. A *ubiquitin* isogene showed the best gene expression stability as a single reference gene, while a 3-gene combination of another *ubiquitin* and two *cyclophilin* isogenes was the best reference gene combination. Comparison between the qRT-PCR and in-house microarray data on roots demonstrated reliability of the identified reference genes to monitor the differential expression of drought-related candidate genes.

**Conclusions:**

Specific isogenes from among the regularly used gene families were identified for use in qRT-PCR-based analyses for gene expression in studies on drought tolerance in rice. These were stable across variables of treatment, genotype, tissue and growth stage. A single gene and/or a three gene set analysis is recommended, based on the resources available.

**Electronic supplementary material:**

The online version of this article (doi:10.1186/s12284-016-0104-7) contains supplementary material, which is available to authorized users.

## Findings

Real-time quantitative reverse transcription PCR (qRT-PCR) is widely used to assess the status of gene expression under variable developmental and environmental conditions (Gachon et al. 2004). Variables for transgene expression (Kohli et al. 2006) also necessitate its analysis by qRT-PCR (Trijatmiko et al. 2016). For the results to be dependable, selection of reference genes that express consistently across the tissue, genotype and developmental stage variables is critical (Wong and Medrano 2005; Guénin et al. 2009). Unless a stably expressed gene is used as a reference, expression level in samples are overestimated or underestimated, and identification of differential expressions is prone to errors. In general, ubiquitously expressed genes that would maintain consistent expression are used. However, these have not been thoroughly tested to be so under different variables. Also, the reference genes generally belong to a gene family and isogenes can be rather variably expressed in different tissues of different genotypes.

In rice, several housekeeping genes have been examined on various samples under different conditions but mostly at seedling stage (Kim et al. 2003; Jain et al. 2006; Narsai et al. 2010; Moraes et al. 2015). Consistent reference genes for rice under drought stress at both, the seedling and reproductive stage are not known. Earlier studies with genes such as the *actin*, *ubiquitin*, *ubiquitin-conjugating enzyme E2* and *eukaryotic elongation factor 1*α (Moumeni et al. 2011; Sharoni et al. 2012; Minh-Thu et al. 2013; Campo et al. 2014) suffer from lack of information on isogene analysis.

An accurate gene expression analysis is central to obtaining insights on adaptation mechanisms to drought stress. Plants regulate the expression of many genes to adapt to water deficit conditions (Shinozaki and Yamaguchi-Shinozaki 2007). A number of drought stress responsive genes were analyzed (Deyholos 2010; Alter et al. 2015), and genes contributing to drought tolerance were suggested (Bhatnagar-Mathur et al. 2008; Osakabe et al. 2014). Drought transcriptome studies in various plant species revealed a largely common response involving similar pathways and genes but again these were largely confined to studies on leaves at seedling stage drought in genotypes known as models for drought tolerance (Lenka et al. 2011; Minh-Thu et al. 2013).

We have redressed the situation by identifying reference genes for relative quantification of transcripts in rice under drought studies, taking into consideration tissues, genotypes and growth stages. Candidate reference genes were pre-screened from public transcriptome microarray datasets by a novel systematic strategy, and then experimental evaluation of the candidates was conducted using qRT-PCR and a rigorous statistical analysis. The best reference genes identified were validated comparatively between qRT-PCR and microarray data.

In principle, reference genes should be independent of any biological response to the treatment under study e.g. drought stress in our case. Good reference genes can be screened from large-scale transcriptome data but the effectiveness of this approach would depend on the depth and breadth of the transcriptome experiments, which almost never simultaneously addressed the variables of treatment, genotype, growth stage and tissues. Thus, in the publicly available data sets, each of which lack addressing one or more of these variables, we focused on the four gene families i.e. *glyceraldehyde-3-phosphate dehydrogenase* (*GAPDH*), *actin, ubiquitin* and *cyclophilin*, that have been recurrently used as reference genes in various organisms. Any additional potential candidate reference genes or gene families would have to be validated in various organisms. Hence, there was value in our conservative approach of considering isogene expression differences within and among the tried and tested four gene families, under the variables of treatment, genotype, tissue and growth stage. Moreover, since reference gene combinations have been recommended (Vandesompele et al. 2002), our effort was to come up with a combination of genes expressed stably under different variables to provide a strict and dependable reference parameter.

**Fig. 1:**
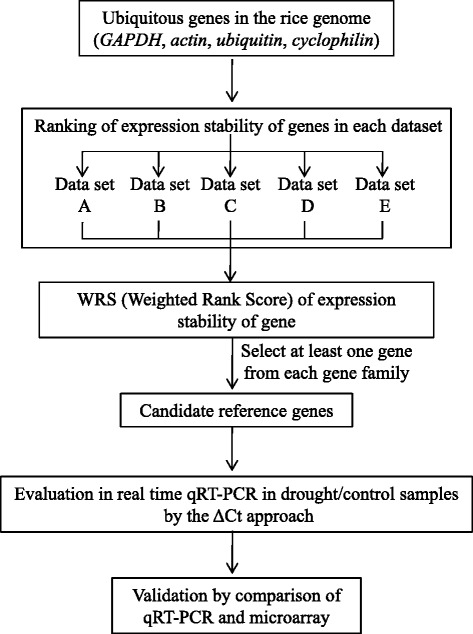
Schematic workflow used for reference gene identification. Expression of genes from four gene families were mined from five different microarray datasets (definitions of the datasets shown in Additional file 2: Table S1). Determination of each gene’s WRS is described in the Materials and Methods (Additional file 1). Ten candidate genes were selected based on their WRS and tested in qRT-PCR. The stabilities of the genes were then assessed using the ∆Ct approach. The most reliable reference genes were validated by the comparison of qRT-PCR and microarray data

**Fig. 2:**
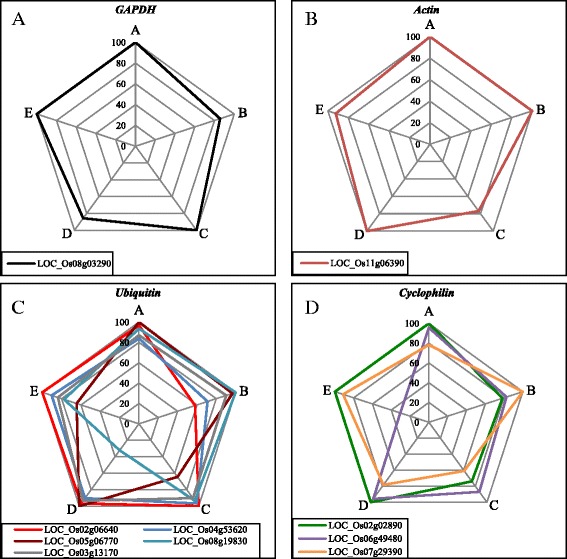
WRS of the reference gene candidates. Radar chart displays the WRS of each reference gene candidate in the five datasets (A to E in each graph). Vertices of the chart represent percentage scores of WRS calculated for genes in each gene family. The highest WRS was adjusted to 100 %. **a** *GAPDH*, **b** *actin*, **c** *ubiquitin* and **d** *cyclophilin*

Seven *GAPDH* isogenes, 13 *actin* isogenes, 31 *ubiquitin* isogenes, and 23 *cyclophilin* isogenes were evaluated based on an expression stability index calculated with the scheme presented in Fig. 1. Microarray data from rice drought tolerance experiments were used to calculate coefficient of variation (CV) of genes, and the CV were then ranked in each gene family (Additional file 1: Materials and Methods; Additional file 2: Table S1). The microarray data were comprised of 11 drought experiments in five different platforms, including data in 10 different growth stages (from vegetative to reproductive stage) and 13 different genotypes (indica varieties: Dagad deshi, IR20, IR64, DK 151, Bala, IR77298-14-1-2-B-10, IR77298-14-1-2-B-13, IR77298-5-6-B-18, 1R77298-5-6-B-11, japonica varieties: Zhonghua 11, Azucena, and Nakdong). The rank was then converted into weighted rank score (WRS), which reflects variety of samples in each microarray data set (Additional file 1: Materials and Methods). Our analysis indicated differential expression of isogenes of the four gene families under one or more variables. Thus, based on the WRS (Additional file 3: Table S2), we selected one *GAPDH* isogene and one *actin* isogene that represented extremely stable expression, five *ubiquitin* isogenes that were moderately stable, and three *cyclophilin* isogenes that were also moderately stable (Additional file 4: Table S3). The expression patterns of the genes selected and their WRS in each data set are shown in Fig. 2 and Additional file 5: Figure S1, respectively.

**Fig. 3:**
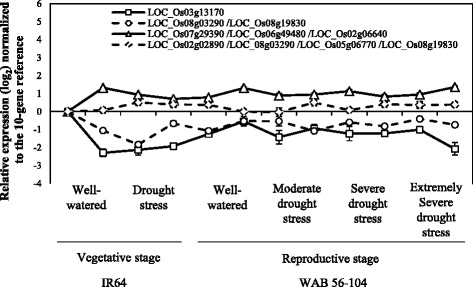
Gene expression patterns of the best different gene/gene combinations. Relative expression level was determined by the 10-gene reference and normalized by the roots of IR64 under well-watered condition. X-axis shows the different samples, and Y-axis shows the expression in log_2_ scale. Error bar represents the standard error for each data point (*n* = 3). R: roots, S: shoots, L: flag leaves

The selected candidate reference genes were evaluated by qRT-PCR with the ΔCt approach. Multiple tissue samples (leaves and roots) from two genotypes (IR64 and WAB 56–104), two growth stages (IR64 in seedling stage and WAB 56–104 in reproductive stage), and stressed at different water availabilities were used for the analysis (Additional file 2). The qRT-PCR products were verified via dissociation curve experiments to see a single product peak for all (Additional file 6: Figure S2). Single genes as well as different gene combinations (up to four genes) were assessed for their expression stability via their mean ∆Ct standard deviation (SD). The top 10 results of single, 2-, 3- and 4-gene categories were tabulated (Table 1). The best single, 2-, 3- and 4-gene references represented constant expression levels generally, but small differences at expression stability were observed (Fig. 3).

**Table 1:** Expression stability of candidate reference genes

Class^a1^	Reference gene	Mean SD of ∆Ct	Rank on expression stability	
			in class^b2^	in total^c3^
Single	LOC_Os03g13170	0.871	1	305
	LOC_Os05g06770	0.929	2	336
	LOC_Os08g03290	0.942	3	342
	LOC_Os06g49480	1.073	4	366
	LOC_Os08g19830	1.093	5	368
	LOC_Os07g29390	1.159	6	373
	LOC_Os11g06390	1.332	7	381
	LOC_Os04g53620	1.561	8	382
	LOC_Os02g06640	1.795	9	384
	LOC_Os02g02890	1.862	10	385
2-gene	LOC_Os08g03290/LOC_Os08g19830	0.714	1	102
	LOC_Os11g06390/LOC_Os08g03290	0.760	2	183
	LOC_Os08g03290/LOC_Os05g06770	0.760	3	185
	LOC_Os08g03290/LOC_Os03g13170	0.773	4	203
	LOC_Os06g49480/LOC_Os08g03290	0.786	5	223
	LOC_Os07g29390/LOC_Os05g06770	0.795	6	239
	LOC_Os07g29390/LOC_Os03g13170	0.827	7	270
	LOC_Os04g53620/LOC_Os08g19830	0.834	8	274
	LOC_Os06g49480/LOC_Os02g06640	0.844	9	281
	LOC_Os03g13170/LOC_Os08g19830	0.856	10	290
3-gene	LOC_Os07g29390/LOC_Os06g49480 /LOC_Os02g06640	0.684	1	49
	LOC_Os06g49480/LOC_Os02g06640 /LOC_Os03g13170	0.686	2	54
	LOC_Os08g03290/LOC_Os05g06770 /LOC_Os08g19830	0.697	3	71
	LOC_Os02g02890/LOC_Os03g13170 /LOC_Os08g19830	0.698	4	75
	LOC_Os06g49480/LOC_Os08g03290 /LOC_Os02g06640	0.699	5	77
	LOC_Os06g49480/LOC_Os02g06640 /LOC_Os05g06770	0.702	6	80
	LOC_Os07g29390/LOC_Os02g06640 /LOC_Os05g06770	0.705	7	86
	LOC_Os02g06640/LOC_Os03g13170 /LOC_Os05g06770	0.708	8	88
	LOC_Os02g02890/LOC_Os05g06770 /LOC_Os08g19830	0.708	9	89
	LOC_Os08g03290/LOC_Os03g13170 /LOC_Os08g19830	0.708	10	91
4-gene	LOC_Os02g02890/LOC_Os08g03290 /LOC_Os05g06770/LOC_Os08g19830	0.618	1	1
	LOC_Os02g02890/LOC_Os08g03290 /LOC_Os03g13170/LOC_Os08g19830	0.621	2	2
	LOC_Os07g29390/LOC_Os06g49480 /LOC_Os02g06640/LOC_Os05g06770	0.622	3	3
	LOC_Os11g06390/LOC_Os02g02890 /LOC_Os06g49480/LOC_Os08g03290	0.626	4	4
	LOC_Os07g29390/LOC_Os06g49480 /LOC_Os02g06640/LOC_Os03g13170	0.627	5	5
	LOC_Os02g02890/LOC_Os06g49480 /LOC_Os08g03290/LOC_Os08g19830	0.627	6	6
	LOC_Os07g29390/LOC_Os02g06640 /LOC_Os03g13170/LOC_Os05g06770	0.630	7	7
	LOC_Os11g06390/LOC_Os02g02890 /LOC_Os08g03290/LOC_Os03g13170	0.631	8	8
	LOC_Os11g06390/LOC_Os02g02890 /LOC_Os08g03290/LOC_Os05g06770	0.634	9	9
	LOC_Os07g29390/LOC_Os06g49480 /LOC_Os08g03290/LOC_Os02g06640	0.639	10	10

^a^1 Category of reference genes

^b^2 Rank in the same number of reference genes

^c^3 Rank among all the reference genes tested

A *ubiquitin* isogene (LOC_Os03g13170) represented the highest stability as a single gene reference. Among the 2-gene combinations, the pair of a *GAPDH* (LOC_Os08g03290) and another *ubiquitin* isogene (LOC_Os08g19830) had the best expression stability. Among the 3-gene combinations, two *cyclophilin* genes (LOC_Os07g29390, LOC_Os06g49480) and yet another *ubiquitin* isogene (LOC_Os02g06640) showed the best expression stability. Finally, among the 4-gene combinations, the set of a *cyclophilin* (LOC_Os02g02890) the *GAPDH* (LOC_Os08g03290) and two *ubiquitin* isogenes (LOC_Os05g06770 and LOC_Os08g19830) was the most stable reference (Table 1). The reason why different ubiquitin isogenes were extracted in the above listed best reference genes could be explained by the idea that most ubiquitously expressed genes showed divergent expressions and a combination of multiple genes minimized such divergence.

**Fig. 4:**
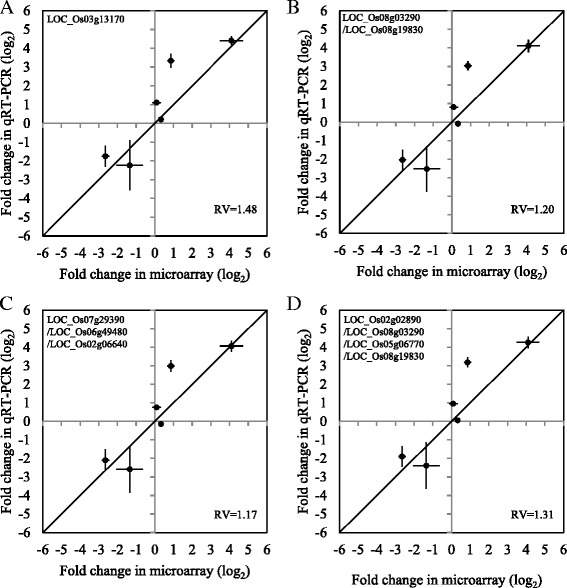
Comparison of qRT-PCR and microarray data of candidate genes from *qDTY*_*12.1*_. Each scatterplot represents a comparison of the qRT-PCR results of the 6 candidate genes normalized using different reference gene combinations. The X-axis displays the fold-change of expression values from control to stressed conditions from the microarray data, while the Y-axis displays the fold-change of expression values from control to stressed conditions in the qRT-PCR. **a** Single reference gene, **b** 2-reference gene combination, **c** 3-reference gene combination, **d** 4-reference gene combination. The RV for each graph was shown at the bottom right corner of each graph. The values for the X and Y- axes are shown in log_2_ scale

In order to validate the best reference gene/combinations above, qRT-PCR results from our previous study (Dixit et al. 2015) were recomputed. Gene expression was quantified using the ∆∆Ct method (Livak and Schmittgen 2001). The expression values were compared to our microarray data (Dixit et al. 2015; GSE78504; http://www.ncbi.nlm.nih.gov/geo/query/acc.cgi?acc=GSE78504). Expressions of eight drought tolerance candidate genes identified in *qDTY*_*12.1*_ were analyzed in roots at seedling stage. We observed a definite trend between the fold-change of expression values in all the four sets of reference genes (Fig. 4). Six out of the 8 genes were observed to have concordance between the qRT-PCR and microarray results after omitting the two outlier data points to reduce their effect on the analysis. The most reliable reference was determined via the goodness of fit to a “y = x” line. This was measured by calculating the residual variance (RV) for each plot, with the RV value being inversely proportional to the concordance between the qRT-PCR and microarray data. Using a single gene reference resulted in an RV of 1.48. The 2-gene combination yielded 1.20; the 3-gene combination, 1.17, and the 4-gene combination, 1.31 (Fig. 4). These results indicated that the degrees of reliability when compared to microarray data were similar in each of the four reference gene approach, but the 3-gene combination offered the best result.

A number of publications exist about particular genes, and various statistical treatments, which can lead to dependable qRT-PCR results (Kozera and Rapacz 2013). Statistical veracity in assessing gene expression by qRT-PCR is an important component of the technique (Vandesompele et al. 2002; Silver et al. 2006). These methods suggested the superiority of multiple reference genes-based analysis to improve qRT-PCR accuracy, and recently Campo et al. (2014) applied a combination of reference genes to monitor dependable expression of their target genes. Utilization of multiple reference genes is a way to relieve the effect of expression variability of single reference genes (Vandesompele et al. 2002). A single reference gene tends to show higher expression variability due to a number of biological factors, but additional reference genes, which are differentially dependent on those factors, could cancel such effects. In this study, we used different combinations of reference genes in order to determine the most reliable gene combination. Based on the mean SD of ∆Ct, the expression stability of the reference increased as more reference genes were used (Table 1). Notably, only the 2-gene and 4-gene combinations have common genes between the different numbers of reference genes used. On the other hand, some single reference genes showed low expression stability. These results indicated the necessity of appropriate reference gene selection for more accurate analysis of gene expression.

Several statistical algorithms have been applied to validate reference genes in plants (Gue´nin et al. 2009; Saha and Blumwald 2014). The algorithms can be classified into two categories: one evaluates absolute Ct values among reference genes such as CV and one-way ANOVA; the other measures pair wise variations of Ct values among genes such as geNorm and the ΔCt approach. The first strategy is robust when mRNA content per total RNA is identical among samples compared, but fails when it differs. The second strategy overcomes this shortcoming, although the estimated gene expression stabilities may include some errors (Guenin et al. 2009). Application of the ∆Ct approach pointed to LOC_Os03g13170 as the most stable reference gene, in accordance with the results from geNorm (data not shown). The reliability of the best reference gene was validated by comparison between the qRT-PCR and the microarray result (Fig. 4).

Jain et al. (2006) documented a *ubiquitin* (*UBQ5*) and *eEF-1*α as the best reference genes to be used. Moraes et al. (2015) reported another *ubiquitin* (*UBQ10*) as the best reference. In the present study, another *ubiquitin* (LOC_Os03g13170) was observed to be the best single reference. However, multiple reference genes showed higher expression stabilities and higher reliability. The best reference gene combinations found in this study, to our knowledge, are not reported yet. These combinations were discovered by exhausting all possible combinations of the candidate reference genes. The best single reference gene was not included in the most stable gene combinations (Table 1). As such, it should not be assumed that combining other genes to a relatively stable reference will automatically yield a better reference.

In conclusion, we identified reference genes for relative quantification of transcript across rice samples under drought conditions, according to their broad expression stability under different variables. Our study indicated that the 3-reference gene combination was the most reliable. Most importantly, some isogenes we identified as broadly stable were different from the ones commonly used as reference genes. This study provides a basis for quantifying gene expression with high accuracy, leading to identification of drought regulated genes, and further leading to better understanding of drought tolerance mechanisms in plants. The identified reference genes are useful to gain insights into regulation of candidate genes against drought stress in rice. In addition, we present an efficient method for screening reference genes and their combinations.

## Abbreviations

Ct, threshold cycle; CV, coefficient of variation; GAPDH, glyceraldehyde 3-phosphate dehydrogenase; mRNA, messenger ribonucleic acid; qRT-PCR, quantitative reverse transcription polymerase chain reaction; RNA, ribonucleic acid; RV, residual variance; SD, standard deviation; WRS, weighted rank score

## Additional files

Additional file 1: Materials and Methods. (PDF 1028 kb) Additional file 2: Table S1. List of publicly available microarray data experiments and their samples used for this study. (XLSX 24 kb) Additional file 3: Table S2. Weighted rank score of gene members of commonly used reference gene families. (XLSX 18 kb) Additional file 4: Table S3. Primers information used in this study. (XLSX 9 kb) Additional file 5: Figure S1. Expression of the 10 selected reference genes in the microarray datasets. (PDF 1031 kb) Additional file 6: Figure S2. Dissociation curves of the reference gene primers products. (PDF 1031 kb)
