# Supplementary material for: Reference genes for accurate gene expression analyses across different tissues, developmental stages and genotypes in rice for drought tolerance
Source: Rice (N Y). 2016 Jul 18;9:32. doi: 10.1186/s12284-016-0104-7 (PMC4949181; doi:10.1186/s12284-016-0104-7)
Supplement: Additional file 1: — Materials and Methods. (PDF 1028 kb) [file 12284_2016_104_MOESM1_ESM.pdf]

Materials and Methods

Analysis of public microarray data

We collected microarray data sets from NCBI Gene Expression Omnibus (Edgar et al. 2002). The data contains 11 sets of rice drought studies, which are comprised of expression profiles in various genotypes, different growth stages from seedling to reproductive stage and different organs under normal and drought stress conditions (Table S1). The data were categorized into 5 sets according to the microarray platform and data normalization status. For the Affymetrix microarray data (platform: GPL2025), raw data (CEL file format) were downloaded, and global normalization was applied via Robust Multi-array Average (RMA) (Irizarry et al. 2003). Probe sets corresponding to only one gene model from the Rice Genome Annotation Project (Kawahara et al. 2013) were selected for the present study. For the NimbleGen Rice 3'-Tiling Microarray (platform: GPL15594 and GPL7344), the data was summarized by calculating the average of probe signals for each gene per chip. For the Agilent microarray data (platform: GPL7252), the data was subjected to per chip quantile normalization by using the limma R statistical package (Ritchie et al. 2015). Log_2-transformed signals were defined as normalized gene expression.

Genes in the 4 gene families, *GAPDH, actin, ubiquitin* and *cyclophilin*, were mined from the functional annotation in the gene model of Rice Genome Annotation Project and the Rice Annotation Project Database (RAP-DB; http://rapdb.dna.affrc.go.jp/). Several genes in these 4 gene families were defined as potential ubiquitous genes in

this study; i.e. 7 of *GAPDH*, 13 of *actin*, 31 of *ubiquitin* and 23 of *cyclophilin*. (Table S2). As an index of gene expression stability, CV of normalized gene expression in a dataset was calculated for each gene using Microsoft Excel. The rank was assigned to genes according to their CV by using the rank function in Excel, with the lowest CV garnering the highest score in each gene family. The ranks were used to screen stably expressed genes across the 5 different microarray datasets by calculating a weighted rank score (WRS), which reflects variety of samples in each microarray data set. WRS in each data set was given by following the equation

$$\mathrm{WRS} = R * (n_g + n_e + n_d + n_o)$$

R denotes the rank in the data set. n_g (number of genotypes), n_e (number of environmental conditions), n_d (number of developmental stages), and n_o (number of organs) were counted in the dataset.

WRS in the 5 datasets were averaged per each gene (Table S2), and it was compared among each gene family members. We assume that a smaller WRS represents a more stable gene expression across microarray data.

Plant Materials

Seeds from *IR64* (*Oryza sativa* L. var. *indica*) were germinated in moist filter paper at 30°C for 5 days. Following germination, these were sown in potted soil and grown in a greenhouse with temperature control under normal lighting conditions in the International Rice Research Institute (Los Baños, Laguna,

14°10'11.81"N,121°15'39.22"E) for 4 weeks from April to May 2015. Drought stress was applied by stopping irrigation for 10 days until leaf rolling was observed and soil moisture content was reduced to approximately 15%. A control treatment comprising of well-watered plants was kept by maintaining enough water content throughout the drought stress. Volumetric soil water content was monitored using ML3 ThetaProbe Soil Moisture Sensor (Delta-T Devices Ltd.). Three biological replicates each for shoots and roots were collected for each treatment. These were frozen in liquid nitrogen and stored at -80°C.

A tropical japonica cultivar *WAB 56-104*, a parental line for NERICA (New Rice For Africa), was grown in a greenhouse of the International Rice Research Institute in 2015. Germinating seeds were planted in a soil of 30 cm height plastic plots in January. When plants were at the booting stage in March, irrigation was stopped, and volumetric soil water content was monitored to prepare samples under progressive drought stress. Roots and flag leaves were collected at 0, 3, 5 and 7 days after initiation of stress. The soil moisture content was 50% (the maximum), 30%, 20% and 15%, respectively. Flag leaves and roots were collected with 3 biological replicates for each treatment. The samples were frozen in liquid nitrogen and stored at -80°C.

RNA preparation

Total RNA was extracted from frozen samples by using the PureLink Plant RNA Purification Reagent (Invitrogen, Thermo Fisher Scientific Inc., USA) as per the manufacturer’s instructions. Total RNA was subjected to DNase I digestion to

eliminate contamination of DNA by using TURBO DNA-free Kit (Invitrogen, Thermo Fisher Scientific Inc., USA). The extracted RNA samples were analyzed using a Nano Drop 2000c spectrophotometer (NanoDrop Technologies), and the absorbance (A) in 230, 260 and 280 nm were measured. RNA samples with A_{260}/A_{280} ratio between 1.9 to 2.1 and A_{260}/A_{230} ratio greater than 2.0 were used for first strand cDNA synthesis and further qRT-PCR analysis.

qRT-PCR assay

For *IR64* samples, 4 μg of total RNA was used for 1st strand cDNA synthesis with oligo $d(T)_{15}$ primer using the ImProm-II Reverse transcription system (Promega Corporation, Madison, USA) according to the manufacturer's instructions. *IR64* cDNA samples were diluted 5-fold and used as a template for each qRT-PCR assay. For *WAB 56-104*, 100 ng and 500 ng of total RNA in roots and leaves, respectively, were employed for 1st strand cDNA synthesis. *WAB 56-104* cDNA samples were then diluted 10-fold and used a template for each qRT-PCR assay.

For *IR64* samples, a 10 μl reaction volume consisted of 1 μl of diluted cDNA, 5 μl of 2X SYBR Select PCR master mix (Applied Biosystems, Thermo Fisher Scientific Inc., USA) and 0.4 μl of 10mM primer for each primer pair. Four μl of diluted cDNA was used for *WAB 56-104*. The reaction conditions followed this protocol: 50°C for 2 minutes, 95°C for 4 minutes, then 40 cycles of 95°C for 10 seconds and 60°C for 30 seconds. Dissociation curve analysis was done for the *IR64* seedling stage samples by subjecting the PCR samples to 60°C to 95°C at 1% temperature ramp speed.

Each assay was carried out with two technical replicates for each biological replicate. Ct values were determined by the 7500 Fast Software v2.3 (Applied Biosystems, USA). The threshold level was automatically set at 10 times of the standard deviation of the fluorescence value of the baseline. Ct values in the same biological replicate were averaged into the representative Ct value, and these were then used for statistical analysis.

Primer information

Transcript sequences of the reference genes assayed were retrieved from the Rice Genome Annotation Project and then used to design gene-specific primers using Primer-BLAST from the NCBI website (Ye et al. 2012; http://www.ncbi.nlm.nih.gov/tools/primer-blast/) with the following parameters: product length of 75-100 bp, melting temperature of 60ºC and GC content of 40 to 60%. Sequence specificity of the primers was tested through a BLAST search against the *Oryza sativa* var. *japonica* sequence.

Statistical analysis by the ΔCt approach

ΔCt approach, documented by Silver et al. (2006), was applied to evaluate gene expression stability. In brief, this method employs average of standard deviation of ΔCt among all the reference genes tested as an expression stability index. In this analysis, we tested plural (up to 4 genes) reference genes. Mean SD of ΔCt was calculated by an in-house Perl script. Geometric mean of Ct values was applied to

calculate ΔCt value of each plural reference gene set as following the method of Vandesompele et al. (2002).

Analysis of microarray data at a seedling stage

We applied in-house rice microarray data generated from *IR64* seedling stage roots under well watered and drought stressed condition (Dixit et al. 2015). The microarray was designed in 56K Agilent rice microarray platform (60K × 8-plex format), which allows monitoring 52,901 rice genes that are corresponding to rice gene model MSU 7.0. The microarray TIF images were processed to run batch extractions by choosing appropriate grid using Agilent's Feature Extraction Software version 11.5.1.1. Raw signal intensities were converted into $\log_2$ expression values, and per chip quantile normalization was carried out for each organ by using limma R statistical package (Ritchie et al. 2015).

Reference for Materials and Methods

Dixit S, Biswal AK, Min A, Henry A, Oane RH, Raorane ML, Longkumer T, Pabuayon IM, Mutte SK, Vardarajan AR, Miro B, Govindan G, Albano-Enriquez B, Pueffeld M, Sreenivasulu N, Slamet-Loedin I, Sundarvelpandian K, Tsai YC, Raghuvanshi S, Hsing YC, Kumar A, Kohli A (2015) Action of multiple intra-QTL genes concerted around a co-localized transcription factor underpins a large effect QTL. Sci Rep 5: 15183

Edgar R, Domrachev M, Lash AE (2002) Gene Expression Omnibus: NCBI gene

expression and hybridization array data repository. Nucleic Acids Res 30: 207-210

Irizarry RA, Hobbs B, Collin F, Beazer-Barclay YD, Antonellis KJ, Scherf U, Speed TP (2003) Exploration, normalization, and summaries of high density oligonucleotide array probe level data. Biostatistics 4: 249-264

Kawahara Y, de la Bastide M, Hamilton JP, Kanamori H, McCombie WR, Ouyang S, Schwartz DC, Tanaka T, Wu J, Zhou S, Childs KL, Davidson RM, Lin H, Quesada-Ocampo L, Vaillancourt B, Sakai H, Lee SS, Kim J, Numa H, Itoh T, Buell CR, Matsumoto T (2013) Improvement of the *Oryza sativa* Nipponbare reference genome using next generation sequence and optical map data. Rice 6: 4

Ritchie ME, Phipson B, Wu D, Hu Y, Law CW, Shi W, Smyth GK (2015) limma powers differential expression analyses for RNA-sequencing and microarray studies. Nucleic Acids Res 43: e47

Silver N, Best S, Jiang J, Thein SL (2006) Selection of housekeeping genes for gene expression studies in human reticulocytes using real-time PCR. BMC Mol Biol 7: 33

Vandesompele J, De Preter K, Pattyn F, Poppe B, Van Roy N, De Paepe A, Speleman F (2002) Accurate normalization of real-time quantitative RT-PCR data by geometric averaging of multiple internal control genes. Genome Biol 3: research0034 doi:10.1186/gb-2002-3-7-research0034

Ye J, Coulouris G, Zaretskaya I, Cutcutache I, Rozen S, Madden TL (2012) Primer-BLAST: A tool to design target-specific primers for polymerase chain reaction.

BMC Bioinformatics 13:134
