# Supplementary material for: Reference genes for accurate gene expression analyses across different tissues, developmental stages and genotypes in rice for drought tolerance
Source: Rice (N Y). 2016 Jul 18;9:32. doi: 10.1186/s12284-016-0104-7 (PMC4949181; doi:10.1186/s12284-016-0104-7)
Supplement: Additional file 5: Figure S1. — Expression of the 10 selected reference genes in the microarray datasets. (PDF 1031 kb) [file 12284_2016_104_MOESM5_ESM.pdf]

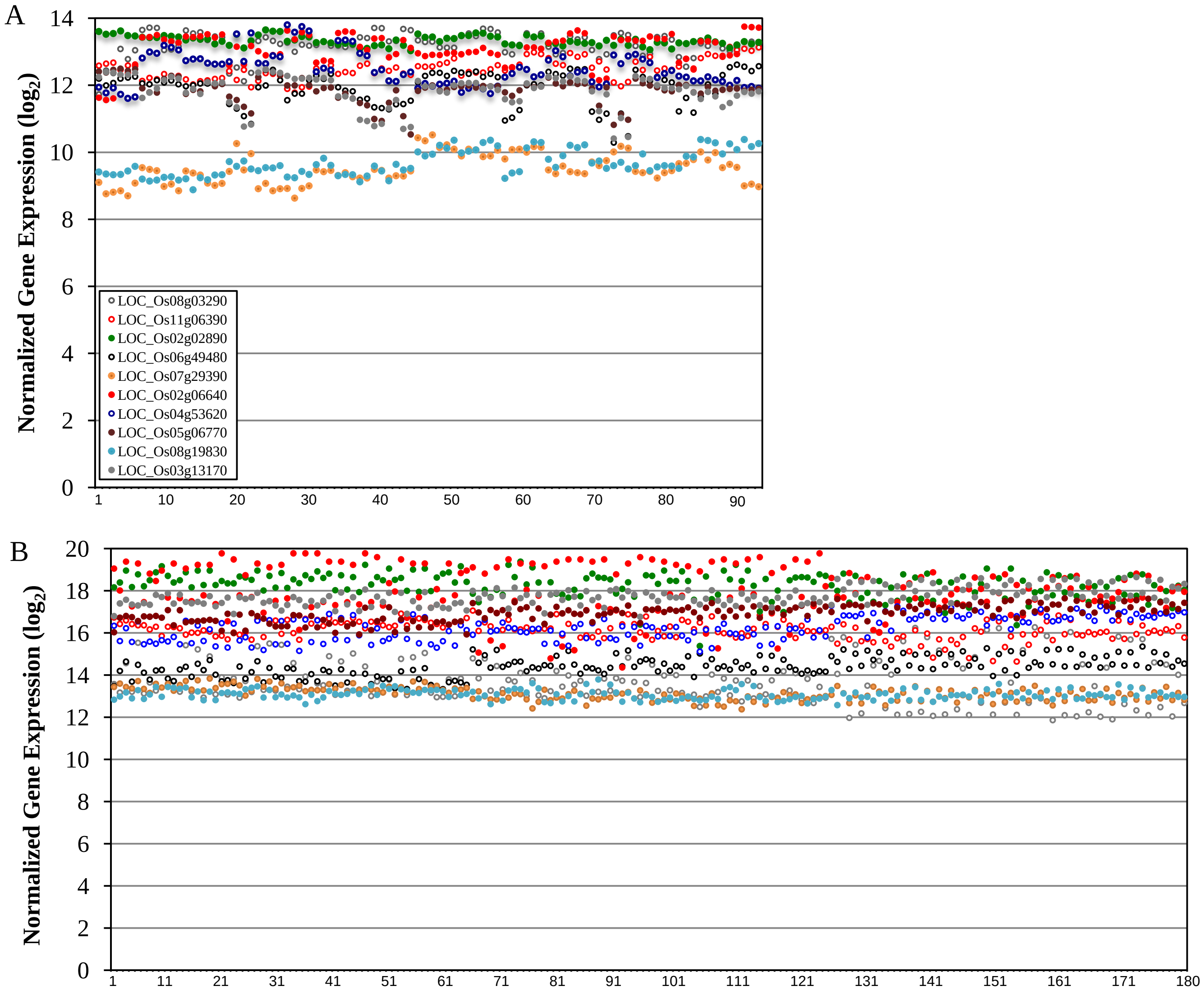

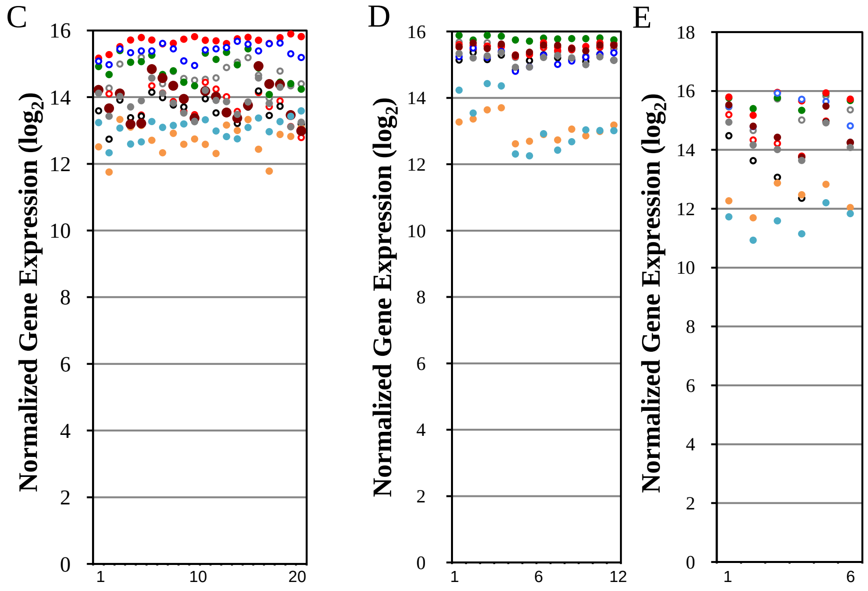

Figure S1. **Expression of the 10 selected reference genes in the microarray datasets.** Gene expressions of the 10 genes selected in this study are plotted as observed in each of the samples used in each of the datasets. The X-axis represents the samples used in each experiment comprising the dataset, while the Y-axis represents the normalized gene expression level (in log_2 scale). Profile identifier is given in the following pages. Each graph represents a dataset: (A) Dataset A, (B) dataset B, (C) dataset C, (D) dataset D and (E) dataset E.

Figure S1

Data set A

1: GSM1020924, 2: GSM1020925, 3: GSM1020926, 4: GSM1020927, 5: GSM1020928, 6: GSM1020929, 7: GSM1020930, 8: GSM1020931, 9: GSM1020932, 10: GSM1020933, 11: GSM1020934, 12: GSM1020935, 13: GSM1020936, 14: GSM1020937, 15: GSM1020938, 16: GSM1020939, 17: GSM1020940, 18: GSM1020941, 19: GSM1525099, 20: GSM1525100, 21: GSM1525101, 22: GSM1525102, 23: GSM1574612, 24: GSM1574613, 25: GSM1574614, 26: GSM1574615, 27: GSM1574616, 28: GSM1574617, 29: GSM1574618, 30: GSM1574619, 31: GSM159259, 32: GSM159260, 33: GSM159261, 34: GSM159262, 35: GSM159263, 36: GSM159264, 37: GSM540080, 38: GSM540081, 39: GSM540082, 40: GSM540083, 41: GSM540084, 42: GSM540085, 43: GSM540086, 44: GSM540087, 45: GSM591761, 46: GSM591762, 47: GSM591763, 48: GSM591764, 49: GSM591765, 50: GSM591766, 51: GSM591767, 52: GSM591768, 53: GSM591769, 54: GSM591770, 55: GSM591771, 56: GSM591772, 57: GSM645322, 58: GSM645323, 59: GSM645324, 60: GSM645325, 61: GSM645326, 62: GSM645327, 63: GSM645328, 64: GSM645329, 65: GSM645330, 66: GSM645331, 67: GSM645332, 68: GSM645333, 69: GSM645334, 70: GSM645335, 71: GSM645336, 72: GSM645337, 73: GSM645338, 74: GSM645339, 75: GSM645340, 76: GSM645341, 77: GSM645342, 78: GSM645343, 79: GSM645344, 80: GSM645345, 81: GSM645346, 82: GSM645347, 83: GSM645348, 84: GSM645349, 85: GSM645350, 86: GSM645351, 87: GSM645352, 88: GSM645353, 89: GSM645354, 90: GSM645355, 91: GSM645356, 92: GSM645357

Data set B

1: GSM755170 Cy3, 2: GSM755170 Cy5, 3: GSM755171 Cy3, 4: GSM755171 Cy5, 5: GSM755172 Cy3, 6: GSM755172 Cy5, 7: GSM755173 Cy3, 8: GSM755173 Cy5, 9: GSM755174 Cy3, 10: GSM755174 Cy5, 11: GSM755175 Cy3, 12: GSM755175 Cy5, 13: GSM755176 Cy3, 14: GSM755176 Cy5, 15: GSM755177 Cy3, 16: GSM755177 Cy5, 17: GSM755178 Cy3, 18: GSM755178 Cy5, 19: GSM755179 Cy3, 20: GSM755179 Cy5, 21: GSM755180 Cy3, 22: GSM755180 Cy5, 23: GSM755181 Cy3, 24: GSM755181 Cy5, 25: GSM755182 Cy3, 26: GSM755182 Cy5, 27: GSM755183 Cy3, 28: GSM755183 Cy5, 29: GSM755184 Cy3, 30: GSM755184 Cy5, 31: GSM755185 Cy3, 32: GSM755185 Cy5, 33: GSM755186 Cy3, 34: GSM755186 Cy5, 35: GSM755187 Cy3, 36: GSM755187 Cy5, 37: GSM755188 Cy3, 38: GSM755188 Cy5, 39: GSM755189 Cy3, 40: GSM755189 Cy5, 41: GSM755190 Cy3, 42: GSM755190 Cy5, 43: GSM755191 Cy3, 44: GSM755191 Cy5, 45: GSM755192 Cy3, 46: GSM755192 Cy5, 47: GSM755193 Cy3, 48: GSM755193 Cy5, 49: GSM755194 Cy3, 50: GSM755194 Cy5, 51: GSM755195 Cy3, 52: GSM755195 Cy5, 53: GSM755196 Cy3, 54: GSM755196 Cy5, 55: GSM755197 Cy3, 56: GSM755197 Cy5, 57: GSM755198 Cy3, 58: GSM755198 Cy5, 59: GSM755199 Cy3, 60: GSM755199 Cy5, 61: GSM755378 Cy3, 62: GSM755378 Cy5, 63: GSM755379 Cy3, 64: GSM755379 Cy5, 65: GSM755380 Cy3, 66: GSM755380 Cy5, 67: GSM755381 Cy3, 68: GSM755381 Cy5, 69: GSM755382 Cy3, 70: GSM755382 Cy5, 71: GSM755383 Cy3, 72: GSM755383 Cy5, 73: GSM755384 Cy3, 74: GSM755384 Cy5, 75: GSM755385 Cy3, 76: GSM755385 Cy5, 77: GSM755386 Cy3, 78:

GSM755386 Cy5, 79: GSM755387 Cy3, 80: GSM755387 Cy5, 81: GSM755388 Cy3, 82: GSM755388 Cy5, 83: GSM755389 Cy3, 84: GSM755389 Cy5, 85: GSM755390 Cy3, 86: GSM755390 Cy5, 87: GSM755391 Cy3, 88: GSM755391 Cy5, 89: GSM755392 Cy3, 90: GSM755392 Cy5, 91: GSM755393 Cy3, 92: GSM755393 Cy5, 93: GSM755394 Cy3, 94: GSM755394 Cy5, 95: GSM755395 Cy3, 96: GSM755395 Cy5, 97: GSM755396 Cy3, 98: GSM755396 Cy5, 99: GSM755397 Cy3, 100: GSM755397 Cy5, 101: GSM755398 Cy3, 102: GSM755398 Cy5, 103: GSM755399 Cy3, 104: GSM755399 Cy5, 105: GSM755400 Cy3, 106: GSM755400 Cy5, 107: GSM755401 Cy3, 108: GSM755401 Cy5, 109: GSM755402 Cy3, 110: GSM755402 Cy5, 111: GSM755403 Cy3, 112: GSM755403 Cy5, 113: GSM755404 Cy3, 114: GSM755404 Cy5, 115: GSM755405 Cy3, 116: GSM755405 Cy5, 117: GSM755406 Cy3, 118: GSM755406 Cy5, 119: GSM755407 Cy3, 120: GSM755407 Cy5, 121: GSM755494 Cy3, 122: GSM755494 Cy5, 123: GSM755495 Cy3, 124: GSM755495 Cy5, 125: GSM755496 Cy3, 126: GSM755496 Cy5, 127: GSM755497 Cy3, 128: GSM755497 Cy5, 129: GSM755498 Cy3, 130: GSM755498 Cy5, 131: GSM755499 Cy3, 132: GSM755499 Cy5, 133: GSM755500 Cy3, 134: GSM755500 Cy5, 135: GSM755501 Cy3, 136: GSM755501 Cy5, 137: GSM755502 Cy3, 138: GSM755502 Cy5, 139: GSM755503 Cy3, 140: GSM755503 Cy5, 141: GSM755504 Cy3, 142: GSM755504 Cy5, 143: GSM755505 Cy3, 144: GSM755505 Cy5, 145: GSM755506 Cy3, 146: GSM755506 Cy5, 147: GSM755507 Cy3, 148: GSM755507 Cy5, 149: GSM755508 Cy3, 150: GSM755508 Cy5, 151: GSM755509 Cy3, 152: GSM755509 Cy5, 153: GSM755510 Cy3, 154: GSM755510 Cy5, 155: GSM755511 Cy3, 156: GSM755511 Cy5, 157: GSM755512 Cy3, 158: GSM755512 Cy5, 159: GSM755513 Cy3, 160: GSM755513 Cy5, 161: GSM755514 Cy3, 162: GSM755514 Cy5, 163: GSM755515 Cy3, 164: GSM755515 Cy5, 165: GSM755516 Cy3, 166: GSM755516 Cy5, 167: GSM755517 Cy3, 168: GSM755517 Cy5, 169: GSM755518 Cy3, 170: GSM755518 Cy5, 171: GSM755519 Cy3, 172: GSM755519 Cy5, 173: GSM755520 Cy3, 174: GSM755520 Cy5, 175: GSM755521 Cy3, 176: GSM755521 Cy5, 177: GSM755522 Cy3, 178: GSM755522 Cy5, 179: GSM755523 Cy3, 180: GSM755523 Cy5,

Data set C

1: GSM935204, 2: GSM935205, 3: GSM935206, 4: GSM935207, 5: GSM935208, 6: GSM935209, 7: GSM935210, 8: GSM935211, 9: GSM935212, 10: GSM935213, 11: GSM935214, 12: GSM935215, 13: GSM935216, 14: GSM935217, 15: GSM935218, 16: GSM935219, 17: GSM935220, 18: GSM935221, 19: GSM935222, 20: GSM935223

Data set D

1: GSM362291, 2: GSM362292, 3: GSM362293, 4: GSM362294, 5: GSM362295, 6: GSM362296, 7: GSM362297, 8: GSM362298, 9: GSM362299, 10: GSM362300, 11: GSM362303, 12: GSM362342

Data set E

1: GSM790485, 2: GSM790486, 3: GSM790487, 4: GSM790488, 5: GSM790493, 6: GSM790494
