# Supplementary figures and images for: Reference genes for accurate gene expression analyses across different tissues, developmental stages and genotypes in rice for drought tolerance
Source: Rice (N Y). 2016 Jul 18;9:32. doi: 10.1186/s12284-016-0104-7 (PMC4949181; doi:10.1186/s12284-016-0104-7)

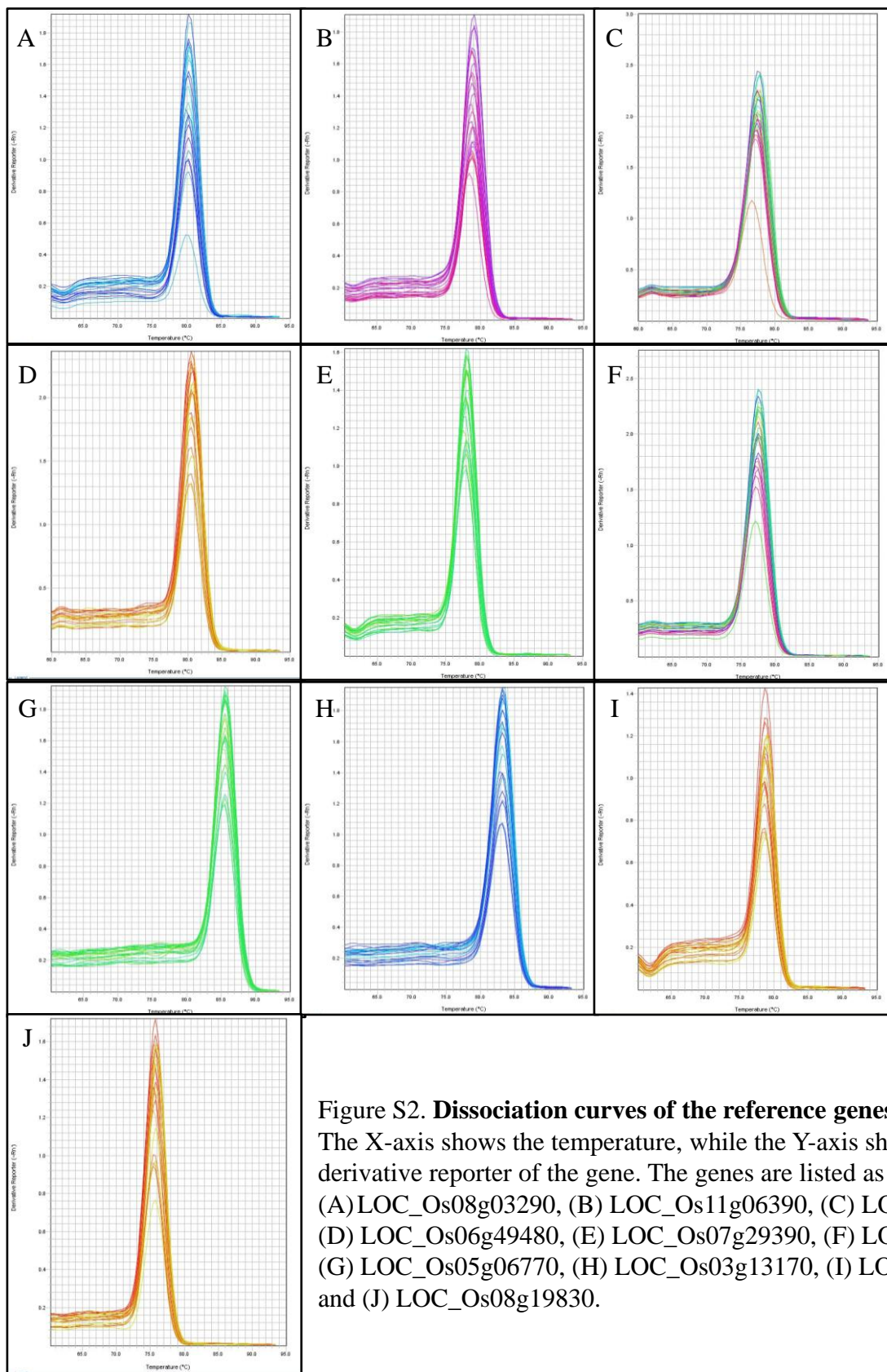

Figure S2

Supplement: Additional file 6: Figure S2. — Dissociation curves of the reference gene primers products. (PDF 1031 kb) [file 12284_2016_104_MOESM6_ESM.pdf]
